# Supplementary material for: Reassessment of Reliability and Reproducibility for Triple-Negative Breast Cancer Subtyping
Source: Cancers (Basel). 2022 May 24;14(11):2571. doi: 10.3390/cancers14112571 (PMC9179838; doi:10.3390/cancers14112571)
Supplement: Supplementary file 1 [file cancers-14-02571-s001.zip › cancers-1685656-supplementary.pdf]

Supplementary Materials: Reassessment of Reliability and Reproducibility for Triple-Negative Breast Cancer Subtyping

Xinjian Yu, Yongjing Liu and Ming Chen

Table S1. Description of public datasets, downloaded from Gene Expression Omnibus.

Study	Country	Population [a]	Sample size	Platform	Sample preservation [b]
GSE103091	France	TNBC	238	HG-U133_Plus_2	FF
GSE103668	Denmark	TNBC	21	HG-U133_Plus_2	FF
GSE76124	USA	TNBC	198	HG-U133_Plus_2	FF
GSE106977	Spain	TNBC	119	HTA-2_0	FFPE
GSE76250	China	TNBC	165	HTA-2_0	FF
GSE86945	Italy	TNBC	100	HTA-2_0	FFPE
GSE86946	Mexico	TNBC	58	HTA-2_0	FFPE
GSE21653	France	BC	266	HG-U133_Plus_2	FF
GSE26639	France	BC	226	HG-U133_Plus_2	FF
GSE147472	Italy	BC	131	HG-U133_Plus_2	FFPE
GSE134359	Mexico	BC	86	HTA-2_0	FF

[a] TNBC: triple-negative breast cancer; BC: breast cancer; [b] FF: fresh frozen; FFPE: formalin-fixed, paraffin-embedded.

Sample code for reanalyzing results of TNBCtype

```
result <- read.csv("path/to/tnbctype/result.csv", )
coef <- read.csv("path/to/tnbctype/coef.csv")
pvalue <- read.csv("path/to/tnbctype/pvalue.csv")


new_result <- data.frame(sample = result$X, subtype = NA) # data frame to store new subtying results
new_coef <- coef.76124[, -c(2,4)] # remove 2 subtypes MSL and IM
new_p <- p.76124[, -c(2,4)]

for (i in 1:nrow(new_coef)) {
  coef <- new_coef[i, ][-1]
  max_co <- max(coef)
  sec_co <- max(coef[coef != max(coef)])
  index <- which.max(new_coef[i, ][-1])
  if (max_co < 0.1 | new_p[i, ][index+1] > 0.05) {
    # if the max_coef < 0.1 or p value of the sbt with max coef > 0.05
    # consider this subtying unreliable
    new_result$subtype[i] <- "UNS"
  } else if (abs(max_co - sec_co) < 0.05) {
    # if (max_coef - second_coef) > 0.05, consider this subtying unreliable
    new_result$subtype[i] <- "UNS"
  } else {
    new_result$subtype[i] <- names(index)
  }
}

# check which samples were re-subtyped
new_result$subtype6 <- result.76124$subtype
new_result[new_result$subtype6 != new_result$subtype, ]
```
